# Role of Bioactive Constituents of *Panax notoginseng* in the Modulation of Tumorigenesis: A Potential Review for the Treatment of Cancer

**DOI:** 10.3389/fphar.2021.738914

**Published:** 2021-10-27

**Authors:** Ming-Ming Tan, Min-Hua Chen, Fang Han, Jun-Wei Wang, Yue-Xing Tu

**Affiliations:** ^1^ Department of Emergency Medicine, Tiantai People’s Hospital of Zhejiang Province (Tiantai Branch of Zhejiang People’s Hospital), Taizhou, China; ^2^ Department of Critical Care Medicine, Zhejiang Provincial People’s Hospital, Affiliated People’s Hospital of Hangzhou Medical College, Hangzhou, China; ^3^ Department of Rehabilitation Medicine, Zhejiang Provincial People’s Hospital, Affiliated People’s Hospital of Hangzhou Medical College, Hangzhou, China; ^4^ Rehabilitation and Sports Medicine Research Institute of Zhejiang Province, Affiliated People’s Hospital of Hangzhou Medical College, Hangzhou, China

**Keywords:** P notoginseng, anti-cancer drugs, phytochemicals, multi-targeted, secondary metabolites, signaling proteins

## Abstract

Cancer is a leading cause of death, affecting people in both developed and developing countries. It is a challenging disease due to its complicated pathophysiological mechanism. Many anti-cancer drugs are used to treat cancer and reduce mortality rates, but their toxicity limits their administration. Drugs made from natural products, which act as multi-targeted therapy, have the ability to target critical signaling proteins in different pathways. Natural compounds possess pharmacological activities such as anti-cancer activity, low toxicity, and minimum side effects. *Panax notoginseng* is a medicinal plant whose extracts and phytochemicals are used to treat cancer, cardiovascular disorders, blood stasis, easing inflammation, edema, and pain. *P. notoginseng*’s secondary metabolites target cancer’s dysregulated pathways, causing cancer cell death. In this review, we focused on several ginsenosides extracted from *P. notoginseng* that have been evaluated against various cancer cell lines, with the aim of cancer treatment. Furthermore, an *in vivo* investigation of these ginsenosides should be conducted to gain insight into the dysregulation of several pathways, followed by clinical trials for the potential and effective treatment of cancer.

## Introduction

Cancer, especially of the breasts, lungs, prostate glands, and colon, is mostly an age-related disease. About 60% of the cancer cases are reported among 13% of the population aged 65 and above, with compromised immune competence, increased exposure to carcinogens, or defective DNA repair system, leading to tumor suppressor gene abnormalities (Zhao et al., 2017). Chemotherapy has been the cornerstone of cancer treatment for decades; yet, it has proven to be ineffective in lowering cancer mortality rates because of factors like final relapse, cytotoxicity, and a variety of additional side effects (Zhao et al., 2017).

Many natural products with pharmacological properties such as antitumor efficacy, low toxicity, and less adverse effects are being investigated as potential therapeutic options (Graham et al., 2000). *Catharanthus* alkaloids, colchicine, etoposide, and taxol are one of the few known examples of well-known drugs (Shibata, 2001; Yun et al., 2001). In this review, we will focus on *Panax notoginseng* (Burk) F.H. Chen, a medicinal herb belonging to the Panax species that is commonly referred to as “Sanqi” in the local Chinese language. The genus *Panax* is classified into three main species: *Panax notoginseng*, *Panax ginseng* (also known as Asian ginseng), and *Panax quinquefolius* (often known as American ginseng) (Taira et al., 2010), all of which have been widely studied and used as medicine or food for many years. The herb is also known as the “miracle root for the preservation of life”, and it is thought to have significant therapeutic value in the East while also acquiring widespread popularity in the West (Barnes et al., 2004; Helms, 2004).

*Panax notoginseng* (Burk) F.H. Chen is thought to have a variety of therapeutic effects and is used in treating cardiovascular disorders, eliminating blood clots, reducing inflammation, discomfort, and swelling (Xie et al., 2007) as well as having antitumor activity (Konoshima et al., 1999; Yun et al., 2001; Sun et al., 2004; Sun et al., 2010), which may open new frontiers in the treatment of cancer (Liu et al., 2020). In general, Rg3, Rh2, and Rg5 play important roles in antitumor effects. (20S)-Protopanaxadiol is a saponin extracted from *Panax notoginseng* (Xu et al., 2015). It is known to decrease tumor growth and lung metastasis in triple-negative breast cancer xenograft and syngeneic models (Liu et al., 2020). However, rather than employing a complete ginseng extract, researchers are now focused on particular ginsenosides to figure out how ginseng works (Gillis, 1997; Attele et al., 1999; Zhou et al., 2004; Cheng et al., 2005; Buettner et al., 2006; Hofseth and Wargivich, 2007). Each ginsenoside that has been identified has a different effect on pharmacology and is varied in its mode of action due to the structural differences. Rb1, Rh2, Rd, Rh4, and Rg3 are just a few of the most investigated ginsenosides. We will concentrate on those ginsenosides (notoginsenoside R1, ginsenosides Rd, Rg1, Rh4, Rb1, and Rg3) that have been shown to inhibit carcinogenesis by targeting several pathways (Li et al., 2007; Gu et al., 2015; Wang et al., 2018). This review provides a thorough overview of *P. notoginseng’s* ethnopharmacology and phytochemistry. Simultaneously, it demonstrates structural variations among different ginsenosides before moving on to the functional assessment of individual ginsenosides, revealing their respective mode of action, starting with the general bioactive extract of *P. notoginseng*.

## Ethnopharmacology of *P. notoginseng*

In Chinese medicine, *P. notoginseng* is considered a non-toxic herb having a sweet, warm, and somewhat bitter flavor. Unlike many other herbs that have a wide range of uses, *P. notoginseng* has traditionally been utilized for a specific purpose. It has also been recognized as one of the most effective herbs for enhancing blood control and hemostasis, among its many other functions. According to herbal research from the *Compendium of Materia Medica*, “Sanqi is a herb pertaining to the blood phase of Jue Yin and Yang Ming and meridians, and it can heal all blood diseases” (Zhao et al., 2017). The drug was eventually developed for a variety of uses, including pain relief, swelling reduction, stasis removal, and bleeding control, in addition to its widespread use in surgery and traumatology. The following are a handful of the most common ginseng applications as recorded by several encyclopedias. Its use for the elimination of necrotic tissues and granulation has been mentioned in the *Golden Mirror of Medicine* compendium. Similarly, it is used to treat hemoptysis (blood coughing), hematochezia (passing fresh blood by anus), hematemesis (blood vomiting), hematuria (blood in urine), and other types of bleeding in the *Compendium of Traditional Chinese and Western Medicine* (Sun et al., 2010).

## Phytochemistry of *P. notoginseng*

Phytochemical studies of the flower, leaf, and stem of *P. notoginseng* have been carried out. Over 200 compounds have been identified from *P. notoginseng*, including volatile oils, polysaccharides, amino acids, flavonoids, aliphatic acetylene hydrocarbons, saponins, cyclopeptides, phytosterols, dencichine, fatty acids, and trace elements (Wang et al., 2016). Saponins, on the other hand, are a significant ingredient responsible for a variety of pharmacological activities. More than 59 saponins have been found in literature, including notoginsenosides, gypenosides, ginsenosides, and flavonoids (Wang et al., 2006). These comprise ginsenosides, notoginsenosides, and gypenosides. Ginsenosides Rg1, Rb1, and Rd and notoginsenoside R1 are the key components of *P. notoginseng* (Wang J.-R. et al., 2014)*.* Dammarane-type and ocotillol-type ginsenosides have also been reported to having been found in this plant (Liu et al., 2020). Other ginsenosides include Re, Rg2, Rh1, and gypenoside. Rg1 and Rb1 make up for more than 20% of the total saponins in *P. notoginseng* (Choi, 2018). Some saponins specific to *P. notoginseng* are R1, Rt, R2, R3, R4, and R6, with the total saponin content estimated to be approximately 15% (Kennedy and Scholey, 2003). Although there are plenty of active compounds present in the species of *P. notoginseng,* the current review particularly focuses over the constituents of saponins.

## Chemical Structure and Classification

**FIGURE 1:**
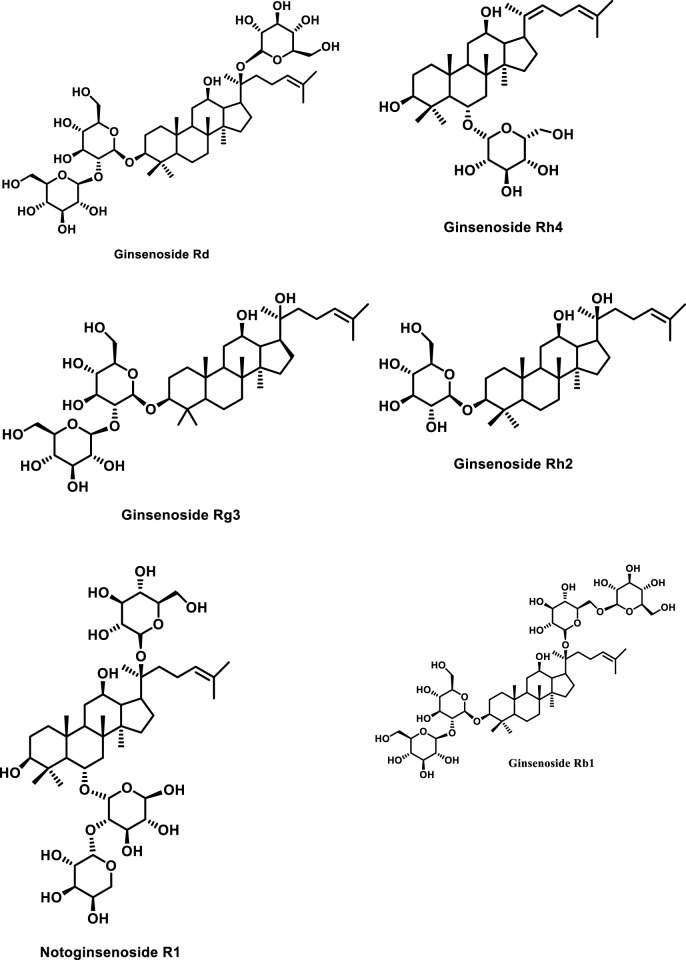
Structures of representative ginseng saponins. PPD, protopanaxadiol; PPT, protopanaxatriol.

The primary pharmacologically active components of *P. notoginseng* are notoginsenosides, also known as notoginseng saponins. Ginsenosides have a steroidal framework with four rings and sugar moieties attached (Nah, 1997; Cheng et al., 2005). Korean ginseng has been shown to contain over 200 compounds, including peptides, ginsenosides, polyacetylenes, polysaccharides, and amino acids (Attele et al., 1999). More than a hundred chemicals have been identified from American ginseng and around 200 active compounds from notoginseng (Xu et al., 2019). Among the isolated products from notoginseng, the predominant and unique components are the dammarane-type ginseng saponins (ginsenosides) (Lu et al., 2009), including R1, Rg1, Re, Rb1, Rd, Rc, Rb2, and Rb3 (Wang P. et al., 2014). All these occur in the main root, rhizome, branch root, and fibrous root with varying percentages. Furthermore, protopanaxadiol and protopanaxatriol genera are two types of dammarane saponins (Sun et al., 2010). In the case of the protopanaxadiol type, sugar at C-3 and/or C-20 positions has OH with sugar moieties attached to it whereas in the other category (i.e., the protopanaxatriol type) the sugar moieties have been found attached with OH at C-3, C-6, and/or C-20 positions (Kim, 2012). Minor components are continually being separated from these ginsengs because of technological breakthroughs. Ginsenosides Rd, Rg1, Rb1, Rg3, and notoginsenoside R1 are the most common saponins found in *P. notoginseng* (as presented in Figure 1) (Sun et al., 2004; Liu et al., 2009).

## Antitumor Activity by the Extract from *P. notoginseng*

Under this section, we would discuss about the general antitumor activity of *P. notoginseng* extracts. The extracts including that of root, rhizome, flower, and berry were used to check the anti-proliferative activities of *P. notoginseng*. *P. notoginseng* extracts were found to be able to arrest human colorectal cancer SW480 cells in the S (synthesis) and G_2_/M phases at the same time, inducing apoptosis. The flower extract was found to have a higher anti-proliferative impact than the extracts from the other parts (Beamer and Shepherd, 2012). Furthermore, the fermented broth of *P. notoginseng* had anti-proliferative effects on the hepatoma Hep3B cells, showing decreased tumor weight and volume. Apart from that, *P. notoginseng* extracts were discovered to increase the effects of chemotherapeutics while reducing the dosage required to obtain the desired results.

### Effect of the Steamed Root of *P. notoginseng* on Anti-Cancer Activities

A human colorectal cell line SW480 was treated for 48 h with an alcohol extract of unsteamed *P. notoginseng* root at a concentration of 50–400 μg/ml, as given in a study published in 2010, but it did not show any anti-proliferative activity (Sun et al., 2010). In contrast, the IC_50_ values of the root extract after 6, 4, 2, and 1 h of steaming were 127.1, 123.7, 131.4, and 259.2 μg/ml, respectively. The anti-cancer activities of *P. notoginseng* root were shown to be greatly enhanced by steaming. To pinpoint the individual constituent responsible for anti-proliferation, Rg1 and Rb1, the predominant constituents of the unsteamed root in addition to Rg3 (that is, the major constituent of the steamed root) were tested on human colorectal cells. Rg3 suppressed the proliferation of more than 99 percent of SW480 cells at a concentration of 300 M; whereas, Rb1 and Rg1 at the same concentration only inhibited the growth of 5% of SW480 cells. Later, it was discovered that the unsteamed root contained 0.33 mg/g of Rg3 but after 6 h of steaming, Rg3 increased to 9.19 mg/g, indicating significant antitumor efficacy. Tang et al. (2018) reported that Rg3 can suppress colorectal cancer cell growth. This is through alteration in the tumor microenvironment by constraining angiogenesis and strengthening antitumor immunity; Rg3 also suppresses liver cancer by decreasing the expression of Na+/H+ exchanger 1 (Li et al., 2018). In addition, the suppression of EGF, ERK, and hypoxia-inducible factor 1 also attenuates hepatocellular carcinoma. Meng et al. (2019) studied the inhibition impact of Rg3 on melanoma and found that it suppresses angiogenesis. The proliferation and metastasis of thyroid cancer is also inhibited by Rg3, as evident from *in vivo* and *in vitro* studies (Wu W. et al., 2018). The viability and self-renewal ability of glioblastoma cells are also inhibited by this ginsenoside (Ham et al., 2019). Chen et al. found that 20(S)-25-methoxyl-dammarane-3β,12β,20-triol (25-OCH3-PPD) shows action against lung cancer cells and androgen-dependent prostate cancer cells. This effect is divulged as being responsible for reducing survival, proliferation, and apoptosis induction (Chen et al., 2016). Wu et al. (2011) detected that 25-OCH3-PPD produced a significant inhibitory effect on activated hepatic stellate cell line t-HSC/Cl-6. Besides, 25-OCH3-PPD, 20(R)-dammarane-3β,12β,20,25-tetrol (25-OH-PPD) can also curtail cell proliferation, inhibit tumors, cause arrest of cell cycle, and prompt apoptosis in cancer cells (Wang et al., 2008). Rg5 causes the apoptosis of breast cancer cells via curtailing of the PI3K/Akt pathway (Liu and Fan, 2018).

### Effect of *P. notoginseng* Broth on Hepatoma

*P. notoginseng* was utilized as a medium for lactic acid bacteria (LAB) fermentation at 37°C for 24 h in a study published by Chiang et al., 2010 (Lin et al., 2010) to examine the effects of the fermented broth on anti-hepatoma activity. The same experiment was also carried out in MRS (well-known LAB media). The total bacterial count in *P. notoginseng* and MRS was found to be 108 cfu/ml and 109 cfu/ml, respectively, suggesting that the herb does not possess any anti-bacterial component.

The viability of the Hep3B cell line was tested by treating it with 500 μg/ml of *P. notoginseng* and MRS containing LAB-fermented broth. Hence, it was noted that *P. notoginseng* –treated broth accounted for 2.2% viability of Hep3B cells, whereas the same amount of MRS-treated broth yielded >100% viability of the cells. These results demonstrate the anti-hepatomal characteristics of *P. notoginseng*.

## Antitumor Effects Induced by *P. notoginseng* Saponins (PNS)

It has been reported that extracts from *P. notoginseng* displayed activity against different carcinomas but, as obvious from earlier references, higher doses of such extracts are required to eliminate cancerous tissues, reducing the tumor’s volume. Under this section, we have considered a very specific category of the extract known as saponins. It has been reported that PNS possesses anti-metastasis characteristics inhibiting the proliferation of cancerous cells. It has also been noted that PNS have markedly cytotoxic effects on the proliferative activity of human colon cancer cell line LoVo in a time- and dose-dependent manner arresting the cell cycle at the S phase (He et al., 2012). PNS have also been accountable for the inhibition of the colorectal cell line (SW480) during the S phase while significantly enhancing the expression of cyclin A and promoting apoptosis (Wang et al., 2007).

Moreover, on assessment of the combinatorial role of PNS along with anti-cancer therapeutics such as cisplatin, a significant increase in the cytotoxicity profile has been observed, in a dose-dependent manner, toward HeLa cells (Yu et al., 2012).

### Effect of PNS on Breast Cancer

Wang J.-R. et al., 2014 reported the viability of 4T1 cells (mammary carcinoma cell line). The cultured 4T1 cells with different concentrations of PNS, ranging from 50 to 400 µg/ml, were incubated for 48 h along with the control. It was observed that no change in cell viability persisted over the concentration of 50 μg/ml, whereas about 75, 60, 30, and 10% reduction in 4T1 viability was observed at 400, 300, 200, and 100 μg/ml concentrations, respectively. Similarly, assays for apoptosis showed a dose-dependent increase in the number of apoptotic cells. After a series of experiments, it was concluded that a low concentration of PNS may induce low-degree apoptosis without rendering any effect on proliferation, whereas high PNS doses, in addition, induce significant apoptosis but also hamper cell proliferation, enhancing cell cycle arrests.

A significant decrease in the migratory effect of 4T1 cells at the dosage of 50 μg/ml was observed, whereas maximal decrement in migration was reported at 200 μg/ml of PNS treatment. Gene expression analysis indicated inhibitory effects of PNS over invasion and migration which in turn are regulated by epithelial to mesenchymal transition (EMT). According to the available literature, the enhanced expression of mesenchymal marker vimentin and loss of E-cadherin expression is potentially regarded as beneficial for tumorigenesis. After incubation with various doses of PNS, it was evident that the mRNA expression of cadherin was significantly upregulated whereas the expression of vimentin was found to get downregulated which supports the notion of an inhibitory effect of PNS over 4T1 cell invasion and migration.

### Effects of PNS on Liver Regeneration via the PI3K/AKT/mTOR Proliferation Pathway

A study conducted by Zhong et al. (2019) revealed that PNS can promote the proliferation of primary mouse hepatocytes in a dose-dependent manner. This was tested by using PNS at different concentrations from 0.01 to 0.50 mg/ml over primary mouse hepatocytes for 24 h. A gradual increase in primary mouse hepatocyte proliferation was noted after treatment with PNS, peaking at 0.12 mg/ml. Furthermore, it was investigated whether Akt/mTOR in primary mouse hepatocytes gets activated by PNS. To affirm this hypothesis an inhibitor of PI3K/Akt (i.e., LY294002) was added to hepatocytes treated with PNS.

It was noted that the proliferative effects of PNS were hampered by LY294002 (*p* < 0.05) as indicated by Edu staining. Next, the phosphorylated and total protein levels of mTOR and PI3K were assessed after treatment with 0.12 mg/ml of PNS with or without LY294002, by Western blotting. Later, it was found that PNS enhanced the phosphorylation of mTOR PI3K and Akt in primary mouse hepatocytes. The same study also reported that PNS not only plays a role in the regeneration of the liver via phosphorylating PI3K and Akt but it also provides a protective effect by inhibiting cellular apoptosis via phosphorylating Akt, leading to Bax protein phosphorylation.

## Role of Individual Ginsenosides and Their Impact on Different Forms of Cancer

### Involvement of the Ginsenoside Rd in Cervical and Colorectal Carcinoma

As mentioned earlier, ginsenosides have been divided into two main categories, protopanaxadiol-type saponins (PDS) and protopanaxatriol-type saponins (PTS). PDS, such as ginsenosides Rh2, Rg3, and Rs4, have been found to inhibit tumor cell metastasis with the induction of tumor cell apoptosis (Liu et al., 2000; Kim et al., 2004; Popovich and Kitts, 2004; Lee et al., 2005). These along with Rk1 and Rk3 are thought to have anti-proliferative effects against liver cancer cells, SNU449, SNU182, and HepG2 (Toh et al., 2011). Rg1 also inhibits invasion and cell migration in the HepG2 liver cancer cell line (Yu et al., 2015). Rh2 inhibits glioblastoma cells via Wnt/β-catenin signaling modulation (Ham et al., 2019). It also enhances survival time and suppresses tumor growth in mice (Wang et al., 2017). In colorectal cancer cells, it suppresses cell growth (Yang et al., 2016). Among them, the ginsenoside Rd is one of the PDS that were isolated from the roots of *P. notoginseng* (Sun et al., 2006) and has been found to possess the strongest activity in terms of tumor cell inhibition. A study conducted by Yang et al. (2006), investigated the cytotoxic and apoptosis-inducing effects of Rd on the HeLa cell line and human colorectal carcinoma (COLO 205). It curtailed cell proliferation and led to apoptosis. Meng et al. (2019) reported similar results.

#### Effect over Cell Proliferation and Morphology

According to the aforementioned study, the growth of HeLa cells was significantly inhibited in a concentration- and time-dependent manner on treatment with Rd. The IC_50_ value of Rd against the HeLa cell line was determined as 150.5 ± 0.8 µg/ml after 48 h of incubation. Also, the cells treated with Rd showed typical apoptotic features characterized by the appearance of apoptotic bodies, volume reduction, and nuclear fragmentation with dense Kelly fluorescence and chromatin condensation when monitored under a fluorescence microscope (Yang et al., 2006).

#### Effect on Apoptosis Rate and Cell Cycle

After the treatment of HeLa cells for 48 h with the ginsenoside Rd, significant changes were observed in the cell cycle distribution at different concentrations (i.e. 120, 180, and 210 µg/ml). The drug-treated cells were characterized by an increase in the S phase cells and a decrease in the G_0_/G_1_ phase cells in a dosage-dependent manner. However, no obvious changes were detected concerning G_2_/M phase cells. After a 48 h treatment of the HeLa cells with Rd, a hypo-diploid (apoptotic) peak of DNA, characteristic of apoptosis, was observed. The corresponding apoptosis rates were determined as 7.4, 15.9, and 35.8% at 120, 180, and 210 µg/ml of Rd, respectively. In the control group, only a minor cell population (ca. 0.9%) was found to undergo apoptosis (Yang et al., 2006).

#### Effect on the Expression of Bcl-2 and Bax Proteins

The function of proteins like Bax and Bcl-2 is to either enhance or inhibit apoptosis. To assess the role of the Bcl-2 family genes in apoptosis induced by the ginsenoside Rd, the expressions of these two proteins were immunohistochemically evaluated in HeLa cells with the aid of the streptavidin ± biotin complex (SABC). It was found that in comparison with the control cells, the Bcl-2 expression was markedly decreased, whereas the Bax expression was enhanced, suggesting that Rd is capable of downregulating the Bcl-2 protein levels while upregulating the Bax protein expression (Yang et al., 2006).

#### Effect on Mitochondrial Transmembrane Potential

Previous studies have reported that the collapse of mitochondrial transmembrane potential is considered a critical event among all cell types undergoing apoptosis, regardless of the inductive signal. To assess the effect of Rd on the mitochondrial transmembrane, both the control- and drug-treated HeLa cells were stained with Rhodamine 123. It was evident that the mitochondrial transmembrane potential of HeLa cells treated with Rd at concentrations of 180 or 210 μg/ml for 48 h was decreased significantly, indicating that Rd is capable of decreasing membrane potential without altering plasma membrane permeability (Yang et al., 2006).

#### Effect on Cell Viability

Caspase-3, in particular, has been reported to have major involvement in the execution of apoptosis and is characterized as the best effector caspase present downstream of Bax and Bcl-2. It is known that the activation of caspase-3 results in cellular death via the proteolytic degradation of various cellular components. To test the viability of cells treated with Rd, the HeLa cells were first incubated with the cell-permeable peptide NH2-Asp-GluVal-Asp-CHO (DEVD-CHO; 2 µM), a highly specific potent inhibitor of caspase-3 and with RPMI-1640 medium (control) for 4-h (Yang et al., 2006). Then, the cells were exposed for 48 h at different concentrations of Rd, and the viability of cells was assessed with the aid of the MTT assay. It was evident that DEVD-CHO significantly increased the viability of the HeLa cell treated with Rd (tested at doses of up to 240 µg/ml) (Yang et al., 2006). Hence, hindering the inhibition of proliferation induced by Rd declares a prominent role of caspase-3 in apoptosis.

### Role Rh2 in MCF Human Breast Cancer Cells

The use of ginseng saponins as discussed earlier has been characterized for various purposes including liver dysfunction, menopausal disorders, hypertension, and cerebrovascular diseases. Among other types, Rh2 ginsenoside has been reported to possess inhibitory characteristics against a series of cultured cancer cell lines including B16 melanoma cells, Lewis lung cells, HeLa cells, and Morris hepatoma cells (Odashima et al., 1979; Kitagawa et al., 1983a; Ota et al., 1987). In addition, it has also been reported that Rh2 can induce reverse phenotypic transformation (Odashima et al., 1979) and differentiation of F9 teratocarcinoma and B16 melanoma cells in the cultures of Morris hepatoma cells (Odashima et al., 1985; Lee et al., 1996).

Taking this into account, G-Rh2 also plays a major role in cell cycle arrest at the G_l_/S boundary of human hepatoma cells (SK-HEP-1) via selectively inducing the protein expression of p27^kipl^ (Kato et al., 1994). Other research groups have also highlighted the role of Rh2 in the induction of apoptosis through the Bcl-2 insensitive pathway in the SK-HEP-1 cell line cultured in serum-free condition by the activation of caspase-3 protease (Park et al., 1997).

As of our current understanding, the eukaryotic cell cycle is under tight regulation of a coordination system comprising of protein kinase complexes including cyclin and cyclin-dependent kinases as its components (Nigg, 1995; Pines, 1995; Sherr and Roberts, 1995).

Among these cyclins, cyclins E and D are considered essential for progression through the G_1_ phase. As cells enter the G_1_ phase, cyclin D and CDK4 and/or CdK6 complexes are required in advance for transitioning through early phases and cyclin E/CdK complexes through late G_1_–S-phases (Dulic et al., 1993; Matsushime et al., 1994; Morgan, 1995; Ohtsubo et al., 1995). A study conducted by Kim et al., 1999 elucidated the role of Rh-2 against MCF-7 anti-breast cancer cells. The following conclusions were deduced after introducing MCF-7 cells with Rh-2 (Oh et al., 1999).

#### MCF-7 Growth Inhibition by G-RH2 and Cell Cycle Arrest at the G_l_ Transition Phase

The MCF-7 cells were treated with increasing amounts of G-Rh2 to observe dose-dependent inhibition and were scored by using a hemocytometer. An inhibition by 40% and 47% was observed on incubating G-Rh2 with cell line concentration sof 50µM and 100 µM for 48 h. To assess whether perturbing the cell cycle–related events are accountable for such kind of suppressive effects, flow cytometry was applied after staining with propidium iodide (PI) to measure the DNA content and the cell cycle distribution of G-Rh2–treated and G-Rh2–untreated cells. The results presented no effects in the control cells treated with the vehicle but led to a marked accumulation of cells in the G_l_ phase in MCF-7 cells treated with Rh2. This suggests that growth inhibition was the result of a block during the G_l_ phase and that such cells did not enter the S phase.

#### The Effect of G-Rh2 on the Apoptosis of MCF-7 Cells

Oh et al. (1999) observed that G-Rh2 inhibited the proliferation of MCF-7 in a dose-dependent manner. This effect was reversible. Lee et al. (2018) reported that Rh2 causes methylation in genes responsible for immune response and tumorigenesis. This phenomenon not only leads to greater immunogenicity but also inhibits cell growth. Rg3 also caused the apoptosis of MCF-7 cells via the inhibition of NF-κB (Kim et al., 2014).

#### G-Rh2 Increased Intracellular Levels of p21 and Decreased Cyclin D

The expression of cell cycle–regulating proteins at the G_l_/S junction, such as D-type cyclins, cyclin E, Cdk2, Cdk4, and p21 were determined to further assess the mechanism by which G-Rh2 arrests cells in the G_l_/S transition phase via Western immune blot (Oh et al., 1999). The results indicated constant expression levels of Cdk2, Cdk4, and cyclin E; however, a decrement in the level of cyclin D3 (10 µM concentration) by G-Rh2 was observed in a time-dependent manner. Next, G-Rh2 was examined as a potential inducer of Cdk inhibitors. A marked increase in the expression of the Cdk inhibitor p21 protein at a dosage higher than 10 µM was observed after Rh2 treatment. Similarly, significant increment in the binding of p21 and Cdk2 was also very prominent. To our advantage, no effect over the expression levels of tumor-suppressive protein p53 and p27, which are the members of the CIP/KIP family, was displayed.

#### G-Rh2 DownrRegulated Cdk2 and Cyclin E–dependent Kinase Activities

As discussed earlier, Cdk2 kinase activity (activated by binding with cyclin E) is essential for G_l_–S phase progression. As the G_l_/S phase of the cell cycle was perturbed by Rh2, there was no effect over the expression levels of Cdk2 and cyclin E. Hence, to verify that whether G-Rh2 inhibits the Cdk2 and cyclin E–dependent kinase activities, histone H1 was used as a substrate to assess the Cdk2 kinase activity which was found to be significantly inhibited in response to G-Rh2 treatment. At 12 and 24 h of G-Rh2 treatment, 20% and 80% decrease was observed in Cdk2 kinase activity, respectively. Similarly, 80% reduction in the activity of cyclin E–dependent kinase was noted after treatment with G-Rh2 following the time interval of 24 h as compared to the untreated control (Choi et al., 2009). These results demarcate that the suppressive effect of G-Rh2 on the cell growth of MCF-7 cells has been partially due to downregulating the activities of Cdk2 and cyclin E–dependent kinase rather than altering the expression of their proteins.

#### G-Rh2 Inhibited the Phosphorylation of pRb and Increased the Binding of pRb and E2F-1

The genetic makeup of every cell tends to play a vital role in cell cycle phase transitions. The product of the Rb gene known as pRb is an important frontier protein in the G_l_ phase of the cell cycle. A tentative relationship was hypothesized as “whether G-Rh2 link decreased the expression of D-type cyclins is associated with the kinetics between the phosphorylation of the pRb and E2F family of MCF-7 cells.”

Molecular analysis has revealed a remarkable decrease in the pRb levels of the expression and was transformed from the hyper-phosphorylated form at 0 h to hypo-phosphorylated or un-phosphorylated form by G-Rh2. Similarly, a time-dependent decrease in the level of phosphorylation of p130 was observed associated with G-Rh2 treatment. Simultaneously, an immunoprecipitation assay has revealed a strong increase in the association of pRb with the transcription factor E2F-1 and enhanced the binding of p130 and E2F-4 after G-Rh2 treatment whereas undetectable association between pRb and E2F-1 was reported among the untreated control cells, indicating that the release of the E2F family gets inhibited after Rh-2 treatment (Table 1; Oh et al., 1999).

**TABLE 1:** Anti-cancer activities of *Panax notoginseng* compounds, their effects, and mechanism of action.

S.No	Compounds	Effects	Cell-line	Mechanism	Assays/Techniques involved	References
01	Ginsenoside Rd	Anti-cervical and anti-colorectal carcinoma	HeLa COLO-25	Decrease in the G_1_/G_0_ phase while increase in the S phase BCL-2 ↓ Bax ↑ Mitochondria *trans*-membrane potential ↓ caspase-3 ↑	Cell proliferation and viability assay, DNA fragmentation assay, and immunohistochemical analysis	Yang et al. (2006)
02	Ginsenoside Rh2	Human breast cancer	MCF-7	Marked accumulation at the G_1_-phase. Cytostatic growth inhibition cyclin D_3_ ↓ P21 ↑ cyclin E–dependent kinase ↓ CdK2 kinase ↓ Hypo-phosphorylation of pRb	DAPI staining, Western Immunoblot assay, co-immunoprecipitation assay, and immunocomplex kinase assay	Oh et al. (1999)
03	Ginsenoside Rh4	Colorectal carcinoma	Caco-2 HCT-116	Marked increase in the G_1_ phase p21, p53/JNK ↑ cyclin D and CdK4 ↓ Bax, cytochrome C and caspase-3 and caspase-9 ↑ mitochondrial cytochrome C ↓ ROS ↑ Fas ↓	H&E staining, CCK-8 assay, Western blotting, and DCFH-DA assay	Wu Q et al. (2018)
04	Ginsenoside Rb1	Ovarian cancer stem cells	SKOV-3 HEYA8	Caspase-3 ↑ Bmi, Nanog, Oct4 ↓ snail and slug ↓ E-cadherin ↑ PI3K/AKT and ERK1/2 hypo-phosphorylation ABCG-2 and P-glycoprotein ↓ inhibition of Wnt/β-catenin pathway	Western blotting, trypan blue exclusion assay,and β-catenin/TCF reporter gene assay	Deng et al. (2017)
05	Notoginsenoside R1	Colorectal cancer	HCT-116 EA.hy926	Significant decrease was observed in MMP9 integrin 1 ↓ E-selectin ↓ ICAM-1 ↓ TEER ↑	MTT assay and immunoblot assay	Lee et al. (2017)
06	Ginsenoside Rg3	Anti-glioma	U87	Significant expression of β-galactosidase p53 and p21 ↑ cyclin-dependent kinase inhibitor (P21CIP) ↑ reactive oxygen species (ROS) ↑ phosphorylated (AKT) ↑	Annexin V staining, MTT assay, SA-β-galactosidase staining, and Western blotting	Sin et al. (2012)
07	PNS	Anti-breast Cancer	4T1	Cell cycle arrest at the G_0_/G_1_ phase apoptosis ↑ cell migration ↓ E-cadherin ↑ vimentin ↓ Brms1, Mtss1 and Timp 2 ↑	Cell migration assay, cell invasion assay, tumor metastasis assay, and histology and immunohistochemistry quantitative real-time PCR	Wang P. et al. (2014)
08	PNS	Anti-colorectal carcinoma	LoVo	Cell cycle arrest at the S phase. A gene known to inhibit metastasis anti-oxidative properties	DPPH-free radical assay, lactate dehydrogenase assay, and flow cytometry	He et al. (2012)
09	Extract of Panax notoginseng (EPN)	Anti-hepato-carcinoma	Hep3B	Inhibition of proliferation at very high doses	Cell cultivation and viability assay, HPLC analysis, and LC-MS/MS analysis	Lin et al. (2010)
10	Extract of Panax notoginseng (EPN)	Anti-colorectal carcinoma	SW480	Arrests cell cycle in the S phase. Cyclin A expression ↑	Annexin V/propidium iodide assay	Wang et al. (2007)

### Role of the Ginsenoside Rh4 Through the ROS/JNK/p53 Pathway in Colorectal Cancer

Colorectal cancer is one of the malignant types of tumors that have presented itself over the history as a third leading cause of cancer-related cell death, according to World Cancer Report (Wu W. et al., 2018). The cancer is characterized by a low postoperative 5-year survival rate, poor prognosis, high likelihood of recurrence, and high metastatic proficiency (Obenauf et al., 2015). Chemotherapeutic agents are the most common therapy suggested to the colorectal patients; however, these compounds induce multiple side effects. The use of herbal medicine as antitumor agents has reduced the side effects in comparison to other drugs. As of our current context, herbs originating from the genus of Panax have shown promising results in several types of cancer.

Among other characteristics, it has also been noted that ginseng after being exposed to heat processes products (majorly Rg3, Rg5, and Rk1) which majorly exerts anti-proliferative effects on various tumor cell types without any compromised renal or hepatic function or major toxicity in mice (Kim et al., 2013). The ginsenoside Rh4, which by its nature is a rare ginsenoside, is produced via elimination of water at carbon-20 of the ginsenoside Rh1 and deglycosylation of the ginsenoside Rg1. Combined with the one-sugar moiety, the triol-type ginsenoside Rh4 is characterized by the ability to readily solubilize in water than other polysaccharide ginsenosides, facilitating its use in pharmaceuticals.

The modality of programmed death pathways can be categorized into type I (apoptotic pathway) and type II (autophagic pathway) which are closely associated with cancer progression (Hengartner, 2000). Similarly, apoptotic cell death of most permanent tumors is regulated by two major pathways (i.e. the mitochondria-mediated intrinsic pathway and the death receptor–mediated extrinsic pathway). Caspases have been found to play a role in both pathways (Fridman and Lowe, 2003). On the other hand, autophagy is more of an evolutionary mechanism conserved for the self-defence process against anti-thymocyte globulins (ATG) (Mathew et al., 2007). In 2018, a study (Wu Q. et al., 2018) was conducted to assess the role of Rh4 a constituent of *P. notoginseng* saponins in the progression of colorectal carcinoma, and the following interpretations were deduced out of it.

#### Anti-colorectal Effects of Rh4 *In vitro* and *In vivo*

To assess the growth inhibition characteristics of Rh4, the mice were inoculated with Caco-2 cells so that a colorectal xenograft model can be established. The mice treated with 40 mg/kg of CAMPTO (Irinotecan) and 20–40 mg/kg of Rh4 exhibited small tumors than the control group after 30 days of treatment. The body weight, however, in both the cases showed an appreciable difference presenting normal weight in Rh4-treated cells as of lower weight of mice treated with CAMPTO, suggesting that Rh4 exhibits little cytotoxicity and fewer side effects (Wu Q. et al., 2018).

#### Tumor Cell Apoptosis

The hematoxylin and eosin staining of tumor tissues and major organs presented a significant decrease in tumor cells with no obvious organ toxicity. Similarly, a histological analysis revealed no obvious injury such as enlargement of the glomerulus, degenerative changes to the cardiac structure, disordered architecture of the hepatic lobule, or collapse of the alveoli. Furthermore, the CCK-8 assay established colorectal carcinoma cells such as Caco-2 and HCT-116 which were significantly inhibited by Rh4 in a concentration- and time-dependent manner (Wu Q. et al., 2018).

#### Induction of Cell Cycle Arrest at the G_0_/G_1_ Phase by Rh4

To analyze the functional role of Rh4 with its anti-colorectal cancer effect, a flow cytometry analysis was conducted. The analysis revealed that after incubation for 24 h with 240 µM, the cells were found to increase in the G_0_/G_1_ phase by 39.06% in the Caco-2 cell line and 31.05% in HCT-116 cell line, respectively, whereas reduction in the number of Caco-2 cells by 18.87% was observed in the S phase while no appreciable difference in the S phase of the HCT-116 cell line was noted. The expression of cell cycle proteins including cyclin D and cyclin-dependent kinase 4 (CDK4) were further analyzed via Western blotting, and the results showed significant reduction in their expression levels, whereas the levels of p53 and p21 on the other hand were enhanced in a dose-dependent manner (Wu Q. et al., 2018).

To further assess the apoptotic pathway induced by Rh4 (that is,intrinsic or extrinsic), the apoptosis-related proteins were investigated by Western blotting. It was observed that Rh4 treatment leads to the upregulation of the cytosolic cytochrome C, Bax and caspase- 3 and caspase- 9 levels and downregulation of the mitochondrial cytochrome C, indicating the involvement of the intrinsic pathway (Table 1). Similarly, the levels of cleaved-caspase-8 increased inversely proportional to levels of Fas, confirming the participation of the extrinsic pathway. Hence, both pathways have been found in triggering the mechanism of apoptosis (Wu Q. et al., 2018).

#### Activation of ROS/JNK/p53 Pathway by Rh4

Previous studies have revealed that steamed EPN can induce ROS generation, in addition to cell death (Kachadourian et al., 2010). To assess the production of ROS on treatment with Rh4, the levels of ROS were measured in Caco-2 cells. An elevated level of ROS was recorded in a concentration-dependent manner on conducting the DCFH-DA assay after getting treated Rh4 in the Caco-2 cell line. On a further note, the study elucidated the anti-cancer mechanism of Rh4 on the MAPK-p53 signaling pathway.

A heightened expression of phosphorylated JNK and p53 was observed in a dose-dependent manner after treatment with Rh4, but a very minimal effect was noticed over the phosphorylation of ERK and p38 in both Caco-2 and HCT-116 cell lines. To assess the relationship between ROS and JNK/p53, the expression of *p*-JNK and p-p53 was followed by pretreatment with NAC, SP600125, and PFT-α. The results showed the attenuation of JNK and p53 phosphorylation by NAC and SP600125 while an inhibition of the p53 expression in Caco-2 cells, indicating that JNK is a downstream target of accumulated ROS, and p53 serves as a downstream effector in response to activated JNK.

### Role of Ginsenoside Rb1 via the Inhibition of Ovarian Cancer Stem Cells

The discovery of cancer stem cells (CSCs) has changed our view of chemoresistance and carcinogenesis (Dean et al., 2005). CSCs represent a sub-group population that has the capability of self-renewal and differentiation properties imparting resistance to these cells against therapy. As of discussion in-process so far, we have noted different constituents (saponins) of ginseng getting consumed worldwide for treatment purposes (Duda et al., 1999). Saponins obtained from the ginseng extract have been noted to possess potent cytotoxic properties marking them as potential chemotherapeutic agents. Under this section, we will articulate a notable ginsenoside known as Rb1 which constitutes 0.37–0.5% of the ginseng extract (Zhu et al., 2004; Wang et al., 2005). It has been reported that the orally administered Rb1 gets metabolized by the intestinal bacteria to its final derivative 20-O-β-D-glucopyranosyl-20(S)-protopanaxadiol (also called compound K) (Hasegawa et al., 1996). Compound K tends to easily get absorbed and sustains for a longer period in the human body. A study conducted by Wong et al., 2017 (Deng et al., 2017) has revealed the targets of ginsenoside Rb1 in the progression of ovarian cancer. The article presented the following conclusions.

#### Cytotoxic and Anti-proliferative Effects of Rb1 and Its Metabolite Compound K

CSCs have been known to self-renew and grow as non-adherent spheres, and the cytotoxic effects of Rb1 and its metabolite compound K were examined over SKOV-3 and HEYA-8 cancer stem cells. The formation of small spheres was observed on treatment with Rb1 and compound K in comparison to control cells in a dose-dependent manner. Similarly, trypan blue exclusion assay revealed a 1.5-fold increase in cell death for Rb and compound K in SKOV-3- and 1.4-2-fold increase in the cell death of HEYA-8 cell lines, respectively.

In addition, Rb1 and compound K accounted for progression in the rate of apoptosis by 5.9- and 9.6-fold in SKOV-3, whereas 1.6- and 2.9-fold in HEYA-8 by expressing the active caspase-3, suggesting that apoptosis may lead to loss in cell viability. Subsequently, cancer stem cells were treated with 250 nM Rb1 and 125 nM compound K for different periods (0, 24, and 48 h) following up with the expression of CSC markers. Bmi-1, Nanog, and Oct4 were three identified ovarian markers that were significantly depleted in a time-dependent manner with maximal depletion observed at 48 h of treatment (Deng et al., 2017).

#### No Relapse on Rb1 and Its Metabolite Compound K Treatment

As pertaining to our prior understanding, the sphere-forming capability found in CSCs is an indirect representation of viable stem cells, and the experiment further investigated the effects of Rb1 and compound K in the relapse situation. SKOV-3 and HEYA-8 primary spheroids were chosen along with the control vehicle and treated with 250 nM Rb1 or 125 nM compound K and then were dissociated and replated as secondary spheroids. By day 2, the control group started to develop into secondary spheroids, whereas the cells treated with compound K and Rb1 did not. However, their removal from SKOV-3 and HEYA-8 CSCs did not restore the secondary spheroid formation activity of CSCs representing irreversible damage (Deng et al., 2017).

#### Enhancement of Chemotherapeutic Drug Effects and CSC Resistance

As of now, other potential drug candidates and compounds of this genus have been known to produce a synergistic effect in combination with other chemotherapeutics. To investigate this effect of Rb1 and compound K on an individual and combinatorial basis, both compounds were used alone or in combination with two frontline drugs in the treatment of cancer (i.e. cisplatin/paclitaxel). It was found that the drugs with relevant doses of 50 and 100 µM had a mild effect of SKOV-3 and HEYA-8 sphere formation. However, Rb1 and compound K in combination with the same doses sensitized the CSCs toward chemotherapeutics (Deng et al., 2017).

#### Inhibition of the EMT Pathway via Compound K

It has already been noticed that Rb1 upon oral administration gets metabolized into compound K (Hasegawa et al., 1996). Furthermore, the underlying mechanism of compound K was examined to present the whole process being executed at the back end. Emerging evidence has suggested an intricate role of EMT in CSC self-renewal (Polyak and Weinberg, 2009). To investigate this fact, the levels of EMT transcription factors were determined. The results declared that the two major transcription markers (that is, snail and slug) were significantly downregulated on treatment with compound K in the presence of cisplatin and paclitaxel.

Similarly, E-cadherin (an epithelial marker and tumor suppressor) expression was reverted back to its normal state. Hence, the underlying molecular mechanism by which compound K has caused inhibition of snail and slug was investigated. As for earlier literature, the PI3K/AKT and ERK1/2 mitogen-activated protein kinase pathways are known to play a critical for the activation of snail and slug (Jorda et al., 2007; Suman et al., 2014). Thus, in order to validate the role of the aforementioned pathways, the phosphorylated (active) forms of AKT and ERK1/2 were assessed. The addition of compound K led to a decrease in the phosphorylation of PI3K/AKT and ERK1/2 which might account for the reduced expression of snail and slug (Table 1). Furthermore, to justify this hypothesis, a constitutively active construct of AKT (E17K) and MEK1 (CA-MEK1) was used. Both snail and slug expressions were still observed after the addition of compound K. Thus, the role of EMT was affirmed in compound K mechanism (Deng et al., 2017).

#### Compound K Inhibits the Wnt/β-Catenin Signaling Pathway

To this extent, the role that compound K plays in the inhibition of CSC survival has still been ambiguous; to further address this issue, molecular analysis was performed. β-Catenin which has been known to play the forefront role in tumorigenesis or self-renewal is an intriguing characteristic (Fodde and Brabletz, 2007; Chien et al., 2009). Therefore, the degree of expression to which β-catenin was produced was analyzed. A marked decline was noted in β-catenin levels on treatment with compound K. β-catenin has also been found to interact with the T-cell factor (TCF) activating various transcriptional genes, and on treatment with compound K, a decrease in the β-catenin/TCF was quite prominent. Similarly, the expression of ABCG-2 and P-glycoprotein drug transporters that are targets of β-catenin/TCF involved in the efflux of chemotherapeutics was also downregulated in SKOV-3 and HEYA-8 cell lines.

### Activity of Notoginsenoside R1 Against Human Colorectal Cancer

Human colorectal cancer (CRC) is one of the predominant cancer types among the human population. Metastasis follows a common route of intravasation and invasion, moving next to the migration of such cells, and getting circulated and adhered which ends up with extravasation and colonization (Leber and Efferth, 2009). An early phase of metastasis (that is, migration and intravasation) gets defined by the degradation of the extracellular matrix (ECM) accounted for the cell-to-cell contact by metalloproteinases (MMP)) (Valastyan and Weinberg, 2011). In the next phase, next to migration and intravasation, the cancer cells enter into circulation. To induce extravasation in a metastatic organ, the cells in circulation need to cling themselves with endothelial cells. This occurs via molecules with characteristic adhesion properties (that is, intracellular adhesion molecule 1 (ICAM1), E-selectin in endothelial cells, and integrin-1 in tumor cells) (Okegawa et al., 2002; Yates et al., 2014).

Thus, the inhibition of the regulators of expression has been considered an effective strategy for suppressing CRC metastasis (Hsieh et al., 2016). The solution of this issue has also been identified from *P. notoginseng* which is known to contain additional components, mainly consisting of dammarane-type saponins. The saponins are divided into three major types (that is, ginseng saponin, gynostemma glycosides, and notoginsenoside) with a total content of ∼12% found in *P. notoginseng* (Yang et al., 2014; Lee et al., 2017). Notoginsenoside R1 (NGR1), a major constituent of *P. notoginseng*, is regarded to curb neurotoxicity, cardiovascular diseases, osteoporosis, and cerebrovascular diseases effectively (Jia et al., 2014; Yan et al., 2014; Wang et al., 2015; Yu et al., 2016). Earlier studies conducted have revealed the beneficiary role of NGR1 in the prevention and treatment of colon cancer and leukemia (Xu et al., 1991; Wang et al., 2007; Wang C.-Z. et al., 2009).

However, more research studies need to be conducted to assess the regulatory mechanism by which NGR1 acts on CRC metastasis. The article published by Wu et al., 2017 (Lee et al., 2017) has shed some light over the role of NGR1 in the metastasis of human colorectal cancer.

#### Role of Notoginsenoside R1 on Human CRC Cells

The viability of HCT-116 cells in the experiment after treatment with 75, 150, or 300 µM of NGR1 was noted to be approximately the same as that of the control during 48 h of incubation. However, the change in the cells’ viability was observed on increasing the concentration to 500 µM, where NGR1 caused an appreciable reduction (58 ± 7.26%) as that from the control (100%) in the duration of 48 h (Lee et al., 2017).

#### Effects of NGR1 over Migration and Invasion

NGR1 has been found to possess profound effects in the HCT-116 cell line in a concentration- and time-dependent manner at the wound-healing areas. A reduced wound closure rate (17–21%) was observed on treatment of the HCT-116 cell line with 75, 150, or 300 µM NGR1 for 24 or 48 h in comparison with the control group (24–34%). Hence, significant suppression of HCT-116 cell migration was induced by NGR1 (Lee et al., 2017).

**FIGURE 2:**
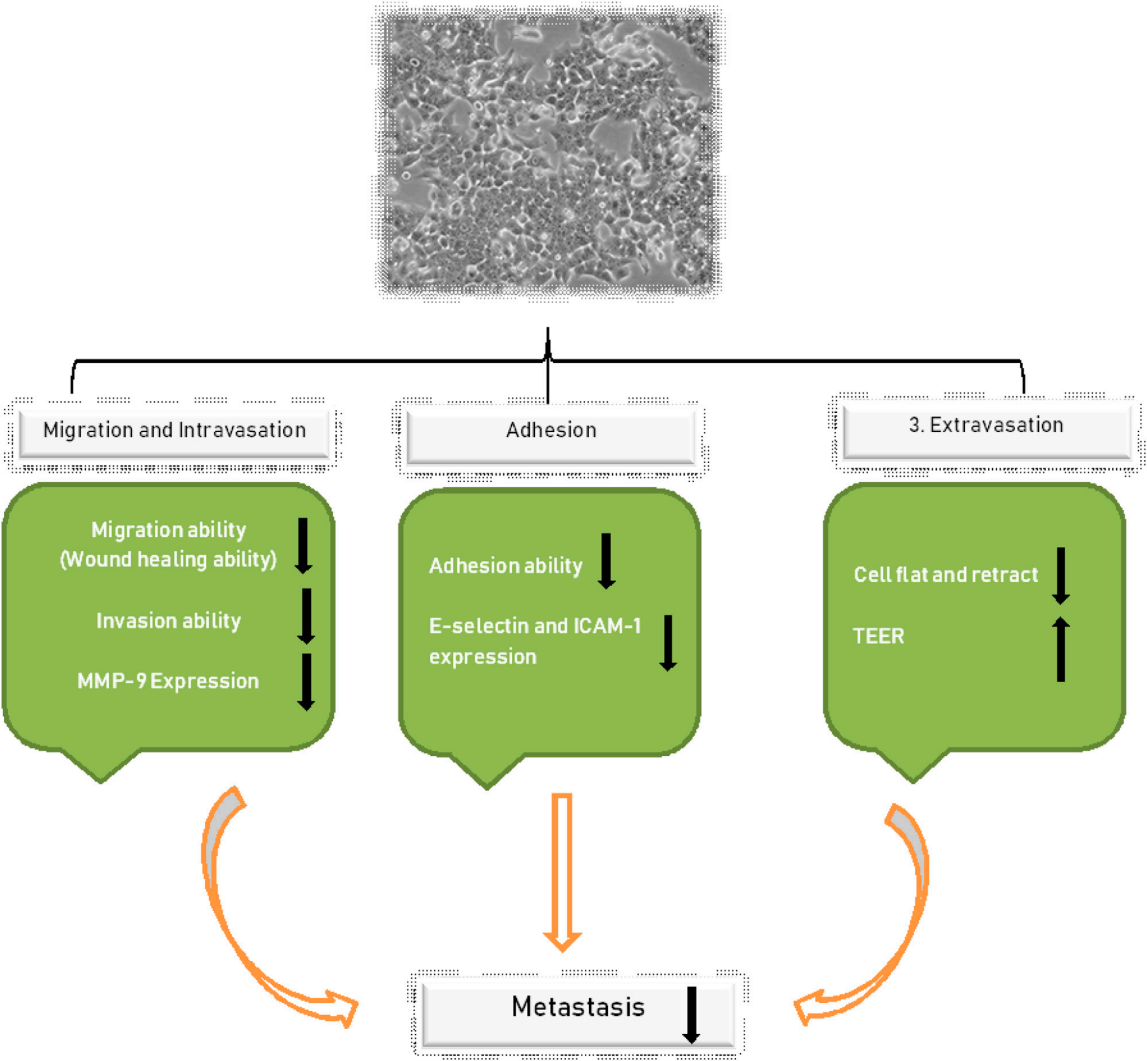
Shows the regulation of NGR1, which controls the migration and intravasation by regulating MMP-9 expression in HCT-116.

Further evaluation was conducted by checking the effects of NGR1 overexpression levels of MMP-2 and MMP-9. The results presented that NGR1 did not regulate MMP-2 expression in the HCT-116 cell line. However, the levels of MMP-9 on treatment with 150 or 300 µM NGR1 for 24 h were significantly decreased by 35 and 68%, respectively, compared with the control group (100%) (*p* < 0.01). The aforementioned results indicate that NGR1 regulates migration and intravasation by regulating the MMP-9 expression in HCT-116 cell lines, as presented in Figure 2 (Lee et al., 2017).

#### Role of NGR1 over the Adhesion of CRC Cells

The property of adhesion among EA. hy 926 cells co-cultured with HCT-116 cells was estimated to get significantly decreased by 18–20% on treatment with 150 or 300 µM of NGR1 for 24 h. To further explore the mechanism, the expression levels of integrin-1 in HCT-116 cells and ICAM-1 plus E-selectin in the EA. hy926 cells were analyzed. The expression levels of all three markers after treatment of HCT-116 cells with NGR1 for 24 h were significantly decreased (Table 1). The level of decrement in comparison to control cells (that is, 100%) was 10–27% in case of integrin-1 and as for E-selectin, the expression levels were 85 ± 5. Similarly, the expression levels were found to be 78 ± 7 and 78 ± 4 after treatment with 75, 150, or 300 µM NGR1 for 24 h in EA. hy926 cells for integrin-1 and E-selectin, respectively (Lee et al., 2017). The corresponding trend of significant reduction with increasing concentration (86 ± 3, 68 ± 3, and 34 ± 2%) was consistent in the case of ICAM-1 compared with the levels in the control cells (100%) (Lee et al., 2017).

#### NGR1 and its Impact over the Extravasation of CRC

To understand the impact of NGR1 on extravasation, changes in HCT-116 cells’ morphology were monitored using scanning electron microscopy. The lipopolysaccharide (LPS)-treated HCT-116 cells reflected a flattened and collapsed appearance, whereas the HCT-116 cells treated in combination with LPS and 300 µM of NGR1 presented a less flattened shape. The study also pursued to monitor the effect of NGR1 on the TEER of EA. hy926 cells. The obtained transepithelial/transendothelial electric resistance (TEER) values were 113 ± 3, 128 ± 14, and 134 ± 16%, after getting treated with 75, 150, or 300 µM NGR1 respectively, signifying an elevation in comparison with the control group (100%). Thus, increase in the TEER value dictates decrement in cell permeability while inhibition of endothelial cells dictates invasion (Lee et al., 2017).

### Role of Rg3 in the Induction of Akt-Dependent Senescence of Glioma Cells

Knowing the fact that somatic cells have a limited intrinsic capacity to divide and reach a non-proliferative state called cellular senescence after a certain period, these cells are also characterized by hypo-responsive behavior toward external stimuli, irreversible growth arrest, atypical gene expression profile, and enhanced β-galactosidase activity with flat and enlarged morphology (Sin et al., 2012). Most tumorigenic cells escape this senescence barrier and escalate to an immortal proliferative state. Therefore, cellular senescence is often regarded as a major tumor suppression mechanism (Vergel et al., 2011). Similarly, therapy-induced senescence represents a functional target that may improve cancer therapy (Schmitt, 2003; Shay and Roninson, 2004; Hornsby, 2007; Ventura et al., 2007).

Under this section, we will discuss the role of Rg3 ginsenoside over a type of cancerous cells known as glioma cells which are primarily located in the brain of adults and are universally considered as fatal. Despite recent advancements in therapy and diagnostics, including radiation, chemotherapy, and surgical resection, the treatment of malignant gliomas remain ineffective due to the high rate of relapse and relative drug resistance (Ohgaki and Kleihues, 2007).

We have noted the role of ginsenosides in various forms of cancer inhibition. Similarly, another ginsenoside 20(S)-ginsenoside Rg3 (20(S)-Rg3) has been reported as an effective medicinal chemical compound with a molecular weight of 784.3 Da molecular weight and C_42_H_72_O_13_ framework (Kitagawa et al., 1983b). Previous studies have reported 20(S)-Rg3 to be safe, and recent evidence from *in vitro* experiments and *in vivo* animal models suggests that 20(S)-Rg3 possesses a diverse range of cancer-inhibitory and anti-mutagenic properties (Helms, 2004; Kim et al., 2004; Jian et al., 2009; Wang B et al., 2009; Kim et al., 2010).

A study conducted by Kim, 2012 reported that senescence-like growth arrest can be caused in U87 glioma cells on treating them with 20(S)-Rg3 at a sub-apoptotic concentration. The following are the results deduced by the aforementioned study.

#### Dose-Dependent Induction of Apoptosis or Senescence in Glioma Cells

The study presents the exposure of U87 glioma cells for 3 days with 20(S)-Rg3 accounted for dose-dependent inhibition. A concentration ≥10 µM was responsible for significant suppression of the growth of glioma cells whereas lesser than that had a little significant impact. Next, the involvement of apoptosis was evaluated in 20(S)-Rg3–induced growth arrest. Annexin V staining revealed that sub-lethal doses (10–20 µM) had a low profile of apoptotic cells in comparison with high doses (50–100 µM) of 20(S)-Rg3.

20(S)-Rg3 (20 µM) was found to completely subdue cell proliferation for at least nine days causing only modest levels of cell death. Glioma cells were treated with various sub-lethal concentrations of 20(S)-Rg3, and the expression of senescence-associated β-galactosidase (SA-β-gal) was measured to assess whether the cell growth arrest in response to 20(S)-Rg3 has been caused due to the induction of cellular senescence (Sin et al., 2012).

The results obtained presented 67% of U87 cells after chronic 20(S)-Rg3 treatment at a 20 µM concentration came up with positive staining whereas only ∼5% cells were stained positive in the DMSO control. Positive staining for SA- β-gal had a characteristic flattened and enlarged appearance that was consistent with cellular senescence. Hence, these findings suggest that senescence as well as apoptosis can be induced by 20(S)-Rg3. In addition, an immunoblot analysis presented an enhanced expression of p53 and cyclin-dependent kinase inhibitor (P21CIP) in 20(S)-Rg3–treated cells (Sin et al., 2012).

#### Triggering Senescence by the Elevation of Reactive Oxygen Species Levels

It is evident from the literature that reactive oxygen species (ROS) are accounted as inducers of senescence. It was evaluated whether the cells treated with 20(S)-Rg3 produces any oxidative stress. ROS levels were assessed via the dichlorofluorescein assay. As it is known that the mitochondrial ROS are majorly responsible for elevated ROS content, therefore, it was also estimated via fluorescence by MitoSOX red as a marker of mitochondrial superoxide. The results showed that the intensity of fluorescence was progressively increased in U87 glioma cells, accounting for the elevated intracellular ROS levels. To verify the results, an ROS scavenger (i.e. N-acetyl-1-cysteine) was added before exposure to 20(S)-Rg3. It was noted that the number of SA-β-gal cells was significantly reduced. These corresponding results strongly suggest the role of ROS in contributing to senescence (Sin et al., 2012).

#### Activation of PI3K/Akt and its Association With ROS Elevation

To examine whether the elevation of ROS has any impact over the PI3K/Akt signaling pathway, the phosphorylated Akt levels of the 20(S)-Rg3–treated U87 cells were measured. U87 cells treated with 20(S)-Rg3 was characterized to have elevated levels of phosphorylated Akt levels at 20 µM concentration. To further assess the role of phospho-Akt in the induction of senescence, siRNA against Akt was employed. The results presented depleted levels of Akt, accounting for decreased SA-β-gal activity compared with the control siRNA cells (Table 1). It has also been reported that ROS generation involves the Akt pathway (Dolado and Nebreda, 2008). Hence, it was investigated that whether elevated ROS levels occur due to the activation of Akt or it is a secondary effect produced by the drug. Transfection with siRNA significantly reduced ROS production by 32%, indicating a vital role of Akt in the regulation of cellular ROS and senescence production (Sin et al., 2012).

## Conclusion

The current review summarizes the usage of *P. notoginseng* and its various constituents in the treatment of cancer. The review has majorly focused on saponins as its pre-dominant component reported against the proliferation of cancer cells as well as an inducer of apoptosis. Six major saponins including ginsenoside Rb1, Rd, Rg3, Rh2, Rh4, and notoginsenoside R1 have been found to significantly upregulate apoptotic markers including caspase-3, Bax, p53, p21, ROS, and TEER, respectively, preventing tumorigenesis. Subsequently, the major pathways accounted for inhibition have also been characterized to present a wholesome layout involved in ginsenoside induced growth arrest. The review serves as a landmark for cancer and molecular biologists to overcome current impediments whereas applying these novel chemotherapeutic leads to the treatment of cancer.
